# Dialysis and pediatric acute kidney injury: choice of renal support modality

**DOI:** 10.1007/s00467-008-0826-x

**Published:** 2009-01-01

**Authors:** Scott Walters, Craig Porter, Patrick D. Brophy

**Affiliations:** 1 CS Mott Children’s Hospital, Department of Pediatrics, Division of Nephrology, University of Michigan, Ann Arbor, MI USA; 2 University of Iowa Children’s Hospital, Department of Pediatrics, Division of Nephrology, Hypertension, Dialysis & Transplantation, University of Iowa, Iowa City, IA USA; 3 Pediatric Nephrology, University of Iowa, 200 Hawkins Dr., Iowa City, IA 52242 USA

**Keywords:** Acute renal failure (ARF), Acute kidney injury (AKI), Pediatric, Peritoneal dialysis, Continuous renal replacement therapy, Dialytic modality

## Abstract

Dialytic intervention for infants and children with acute kidney injury (AKI) can take many forms. Whether patients are treated by intermittent hemodialysis, peritoneal dialysis or continuous renal replacement therapy depends on specific patient characteristics. Modality choice is also determined by a variety of factors, including provider preference, available institutional resources, dialytic goals and the specific advantages or disadvantages of each modality. Our approach to AKI has benefited from the derivation and generally accepted defining criteria put forth by the Acute Dialysis Quality Initiative (ADQI) group. These are known as the risk, injury, failure, loss, and end-stage renal disease (RIFLE) criteria. A modified pediatrics RIFLE (pRIFLE) criteria has recently been validated. Common defining criteria will allow comparative investigation into therapeutic benefits of different dialytic interventions. While this is an extremely important development in our approach to AKI, several fundamental questions remain. Of these, arguably, the most important are “When and what type of dialytic modality should be used in the treatment of pediatric AKI?” This review will provide an overview of the limited data with the aim of providing objective guidelines regarding modality choice for pediatric AKI. Comparisons in terms of cost, availability, safety and target group will be reviewed.

## Introduction

The epidemiology of pediatric acute kidney injury (AKI), the treatment approaches, identification and validation of objective variables and technological refinements in monitoring equipment, access, and renal replacement therapy (RRT) machinery have all changed over the past decade. Overall, AKI remains a significant contributor to the morbidity and mortality of critically ill infants and children, especially those with multi-system organ failure [1]. There have been at least 30 different definitions of AKI reported in the literature [2]. To date, it has generally been accepted that AKI is characterized by the failure of the kidneys to regulate electrolyte, acid–base and fluid homeostasis adequately, with a concomitant reduction in glomerular filtration rate [3, 4]. In practical terms this may be demonstrated in the pediatric patient by an increase in nitrogenous waste products [blood urea nitrogen (BUN)], an associated increase in serum creatinine (> 50% above baseline level) and, in most cases, a concomitant reduction in urine output (less than 0.5–1 ml/kg per hour) [3–5]. These varied definitions have resulted in our inability to assess data sets comparatively between studies, making meta-analysis extremely difficult. This also has significantly hindered any comparative analysis between available RRT options in like patient groups. Controversy remains as to when specifically RRT is indicated in terms of creatinine/BUN rise, fluid overload, associated organ failure, type of RRT to employ, which patient population will benefit most (if at all) from which RRT therapy and how early should therapy be initiated.

So that such fundamental questions can be addressed, a commonly accepted and utilized AKI definition is required. The Acute Dialysis Quality Initiative (ADQI) was formed in early 2000 and was composed of nephrologists and intensivists, including pediatric representation (https://doi.org/www.ADQI.net). The group objectively scrutinized available AKI data and classified it as to scientific merit. From these consensus meetings, criteria for AKI were defined and validated. These criteria are known as the risk, injury, failure, loss, and end-stage renal disease, or RIFLE, criteria [2]. A recent pediatric study has validated a modified form of these criteria, the “pRIFLE” [6].

Epidemiologic distribution of pediatric AKI varies according to the type of institution reporting results. Those units containing high surgical and trauma volumes, as well as concentrated sub-specialty services, may garner a greater number of pediatric patients with AKI than the smaller or less developed centers [7–11]. Recently, Hui-Stickle et al. noted the dramatic broadening of pediatric AKI epidemiology [12]. Prior to the 1990s, the most prevalent causes cited were hemolytic uremic syndrome, other primary renal causes, sepsis, and burns [13–18]. Now, the most common causes in developed countries may involve multiple system diseases or failures, including congenital heart disease, acute tubular necrosis, nephrotoxic medications, and sepsis [12]. In developing countries hemolytic uremic syndrome (HUS) continues to be reported as one of the primary causes of pediatric AKI [19].

In parallel to the changes in AKI epidemiology, there have been dramatic changes to our approaches to RRT in pediatric AKI. The conservative management goals of pediatric AKI include symptomatic measures such as control of fluid balance, dietary and blood pressure management, and improvement of survival time. In the setting of AKI, disturbed fluid or metabolic balance often necessitates the initiation of RRT [14, 20, 21]. Historically, the reported mortality rates for children requiring dialysis ranged anywhere from 35% to 73% [7–10, 22]. However, more recent pediatric RRT demographic data has stratified diagnoses and clarified outcome numbers, suggesting that refinement of variables, use of severity of illness scores, and earlier intervention are, for the first time, providing improved care with improved outcomes [23].

A great number of modalities is currently available for the provision of RRT in the pediatric patient with AKI. Intermittent hemodialysis (IHD), peritoneal dialysis (PD), and continuous renal replacement therapies (CRRT) such as continuous venovenous hemodialysis (CVVHD) (predominantly diffusive clearance), continuous venovenous hemofiltration (CVVH) (convective clearance), or continuous venovenous hemodiafiltration (CVVHDF) (both convective and diffusive clearance), may be employed to provide enhanced solute clearance and ultrafiltration [3, 24, 25]. While temporary IHD is the most efficient modality for fluid and metabolic control, it is often not feasible for our smallest and/or sickest patients in the pediatric intensive care unit (ICU), due to large extracorporeal circuit volumes and the inability of patients to tolerate rapid fluid shifts. In such settings the alternative use of PD or CRRT has become the mainstay of RRT. In general, PD has long been preferred and utilized as a form of continuous therapy in the setting of AKI in pediatrics [26, 27]. The continued improvements and refinements of CRRT technologies has allowed these systems to be employed in our smallest patients with more-complicated and unstable disease, especially in resource-rich countries [28–33]. As more and more providers of pediatric care are equipped with the ability to provide CRRT, it is fast becoming the standard of care in the ICU setting. Warady and Bunchman [34] noted that, in just a 4-year period (1995–1999), the preferred modality for the treatment of pediatric AKI had changed from PD to CRRT, with a 100% (18 to 36) increase in the number of centers reporting CRRT as the primary initial modality of treatment. Currently, it is unclear which patients with AKI require which specific therapy, and some patients may benefit from the provision of PD rather than IHD or CRRT in specific circumstances. Indeed, individual practices or clinical settings may not have the availability of a variety of modalities. The choice of PD, IHD or CRRT is dependent on a variety of factors, both from a patient perspective and from a center perspective. The following will review the current state of literature comparing the use of PD, IHD and CRRT for the treatment of AKI in pediatrics, outlining the advantages and disadvantages in relationship to efficiency, complications, complexity, disease process and outcomes.

### Modality choice

The choice of an appropriate modality for AKI depends on the clinical status of the patient as well as the dialytic indication. Clinically, several important patient conditions require attention. Initial patient assessment should focus on whether multiple organ systems are involved and to what extent. A patient with AKI solely, will predictably have a better potential outcome than those with multi-system failure [23, 35]. If AKI is the only morbidity associated with the patient, then it is important that quantification of urine output (if present) be acknowledged. This will help determine the patient’s ability to handle fluid and solute load. Classically defined indicators for RRT initiation in the setting of AKI are extrapolations of those we have commonly used for end-stage renal disease (ESRD) and include: metabolic/electrolyte imbalance, uremia with bleeding and/or encephalopathy, hypervolemia with pulmonary edema/respiratory failure, intoxications, inborn errors of metabolism (IEM), and nutritional support. While these may be recognized indicators, to date there has been no adequate definition of what “timing of initiation” means. The decision to initiate RRT may be affected by strongly held physician beliefs (in terms of indications), patient characteristics (including age/size, illness acuity, and co-morbidities) and organizational characteristics (including resource availability, type of institution, type of ICU, type of provider, and perceived cost of therapy). All these factors will ultimately determine the appropriateness and availability of modality choice.

## Indications

Specific indications for RRT typically include the need for ultrafiltration (i.e. fluid removal), either for symptomatic volume overload, or to make space for nutrition, medications, and blood product support and/or solute removal (i.e. urea, potassium), either for uremia or for removal of a dialyzable toxin. In addition to these clinical variables, the use of specific modalities in terms of need for nutritional support to aid in patient recovery from AKI or its underlying cause [36–38] must be considered. The rapid removal of solute (urea) and correction of electrolyte abnormalities (particularly elevated levels of potassium) are of extreme importance in the setting of AKI. While PD, IHD and CRRT can rapidly correct hyperkalemia and uremia, IHD and CRRT provide greater clearance of higher molecular weight solutes than PD. The rapidity of solute generation and its particular urgency for removal, as in tumor lysis syndrome, IEM, hyperammonemia, symptomatic hyperkalemia, or ingestion of dialyzable toxins, require IHD or CRRT rather than PD, whereas mild uremia can be treated with any of the modalities [39–43]. Urgent fluid removal required in patients with pulmonary edema and difficulty in ventilating may only be achieved by IHD or CRRT. Mild volume overload can be treated with any modality. For example, a hemodynamically unstable patient with overwhelming sepsis, fluid overload, respiratory compromise, pressor requirements and renal involvement may necessitate initiation of CRRT for close fluid status control, whereas a post-operative cardiac patient with minimal fluid overload and hemodynamic instability may be better served by PD. The physical characteristics of the type of solute to be removed (i.e. molecular size, percentage of protein binding) may determine the need for IHD vs PD vs CRRT (CVVH vs CVVHD). The metabolic status of the patient may also reflect the type of dialytic solution required.

## Patient characteristics/contraindications

The physical condition of a patient in terms of underlying disease process, size, previous surgical procedures and overall stability will often dictate choice of modality. Contraindications to PD may include diaphragmatic hernia, recent intra-abdominal surgery, intra-abdominal sepsis, lack of an adequate peritoneal surface, or intra-abdominal malignancy. Also, in AKI secondary to HUS, gut involvement may be severe enough to preclude PD, as may necrotizing enterocolitis in a neonate. Severe hypotension may prohibit the use of IHD. A patient’s size may prevent successful vascular access or even temporary PD. Indeed, in our smallest neonates, vascular access may not be achievable with double lumen catheters in the neck or groin, and single lumen 5 Fr. catheters may need to be placed in the umbilical vessels. In the same neonatal patient even temporary PD may be impossible when automated machinery is used, due to tubing “dead space”. Such considerations must be taken into account in determining the feasibility of dialysis delivery in our smallest patients. The presence of a coagulopathy may impact on performance of IHD or CRRT, or the ability to establish vascular access for either modality.

## Organizational characteristics

In some geographical locations resources are the limiting factor. PD may have to suffice if no other form of RRT is available. For example, the literature clearly supports IHD and CRRT over PD in the treatment of IEM; however, even PD may provide clearance and should be utilized when no other options exist. Along with availability, the ability for IHD or CRRT to be initiated rapidly may be an issue, depending on staffing and expertise.

The overall approach to the uniform clinical assessment is to determine what the major goal of dialytic therapy is. Does the patient need only solute clearance? If so, what solute (molecular weight), and how quickly? Does he or she need primarily ultrafiltration? If so, how quickly must fluid removal be achieved and what is the hemodynamic stability? What are the resources available? What limiting factors are present in terms of the patient’s condition? All of these considerations must be taken into account if we are to deliver appropriate therapy in the right context.

### The case for PD in pediatric AKI

Historically, PD has been the primary RRT modality employed in pediatric care. Experientially, most clinicians have a relatively greater comfort level with employing this therapy, and it has been effective for the management of childhood AKI [7–10, 44–48]. Indeed, recent reports from Nigeria, India and Brazil have demonstrated its continued efficacy in the treatment of pediatric AKI [19, 49, 50]. PD offers a variety of advantages in the acutely ill pediatric population with AKI (see Table 1).

**Table 1:** Comparison of the advantages and disadvantages of continuous renal replacement therapies (CRRT) and peritoneal dialysis (PD) and intermittent hemodialysis (IHD). *IPD* intermittent peritoneal dialysis, *VP* ventriculoperitoneal, *ICU* intensive care unit

Variable	CRRT	PD	IHD
Continuous therapy	Yes	Yes	No
Hemodynamic stability	Yes	Yes	No
Fluid balance achieved	Yes, pump controlled	Yes/no, variable	Yes, intermittent
Easy to perform	No	Yes	No
Metabolic control	Yes	Yes	Yes, intermittent
Optimal nutrition	Yes	No	No
Continuous toxin removal	Yes	No/yes, depends on the nature of the toxin—larger molecules not well cleared	No
Anticoagulation	Yes, requires continuous anticoagulation	No, anticoagulation not required	Yes/no, intermittent anticoagulation
Rapid poison removal	Yes/no, depending on patient size and dose	No	Yes
Stable intracranial pressure	Yes	Yes/no, less predictable than CRRT	Yes/no, less predictable than CRRT
ICU nursing support	Yes, high level of support	Yes/no, moderate level of support (if frequent, manual cycling can be labor intensive)	No, low level of support
Dialysis nursing support	Yes/no, institution dependent	Yes/no, institution dependent	Yes
Patient mobility	No	Yes, if IPD used	No
Cost	High	Low/moderate. Increases with increased dialysis fluid used	High/moderate
Vascular access required	Yes	No	Yes
Recent abdominal surgery^a^	Yes	No	Yes
VP shunt	Yes	Yes/no, relative contraindication	Yes
Prune belly syndrome	Yes	Yes/no, relative contraindication	Yes
Ultrafiltration control	Yes	Yes/no, variable	Yes, intermittent
PD catheter leakage	No	Yes	No
Infection potential	Yes	Yes	Yes
Use in AKI-associated inborn errors of metabolism	Yes	No	Yes
Use in AKI-associated ingestions	Yes	No	Yes

^a^Omphalocele, gastroschisis, frequent or extensive abdominal surgery

### Technical aspects

From a technical aspect, access can be relatively quickly and safely obtained, even in hemodynamically unstable patients and those with a coagulopathy. PD is superior in its requirement for less clinical expertise, fewer equipment resources, and cost. This dialysis modality is critical to AKI in facilities where pediatric IHD and CRRT are not available, as is the case in more rural areas and most developing countries [19, 50]. PD does not require vascular access, thereby allowing critically ill patients to be dialyzed with preservation of vasculature for future procedural necessities. Access in the form of classically surgically placed Tenckhoff catheters, or short-term PD or adapted PD catheters placed at the bedside percutaneously [51, 52] in patients unable to tolerate surgical placement, allow the rapid institution of therapy. This also potentially eliminates the need for an ICU environment for patients whose condition is stable. This technique is usually well tolerated in our smallest patients. In the USA, the neonatal 5 Fr. temporary “stab” catheter (Cook Medical Incorporated, Bloomington, IN, USA), is no longer available, but the 8.5 Fr. catheter is still available and can be placed in most neonates without difficulty. Drawbacks for the use of short-term PD “stab” catheters include the risk of leakage, infection, and the initiation with relatively small volumes of dialysate (usually 10 ml/kg) limiting adequate clearance. Complications can also include bladder and bowel perforation, along with bleeding issues related to the procedure. If possible, surgical placement is therefore advised [46, 53, 54].

If very low-volume, automated, PD cycler systems are not available, PD can be performed manually in infants. Institution of continuous PD may be easily accomplished with manual exchange systems [the Dialy-Nate system/Gesco Dialy-nate (Utah Medical Products, Midvale, Utah, USA) or the PD-Paed system (Fresenius Medical Care, Bad Homburg, Germany)] for infants. These manual exchange systems are not inexpensive, but they are readily available worldwide, unlike IHD and CRRT equipment. Dialysate is available worldwide and in circumstances where avoidance of lactate-based solutions is in the patient’s best interest (i.e. patients with hepatic dysfunction or lactic acidosis); commercially available pure bicarbonate and bicarbonate/lactate solutions are available outside the USA. Alternately, “custom-made” bicarbonate solutions can be prepared by hospital pharmacies at an additional cost [55, 56]. The dextrose used in PD dialysate can provide an extra source of carbohydrate nutrition and calories [57, 58]; however, this can also lead to hyperglycemia [59], necessitating insulin correction. Supplementation with sodium (if commercial preparations are used) and amino acids may be required, due to increased clearance in the setting of AKI.

### Efficiency and therapeutic advantages/disadvantages

Therapeutically, PD provides gradual and continuous solute clearance and ultrafiltration, thereby mimicking renal function to some extent. This property is a major contributing factor to treatment of pediatric patients with cardiovascular instability with PD. Several retrospective analyses have demonstrated that PD can be successfully performed in pediatric patients with hemodynamic instability and multi-system organ failure requiring vasopressor support [48, 49]. However, for these patients, the efficiency of PD may be suboptimal, and vigilance on the part of the clinician is required. Close monitoring is also required for patients with pre-load-dependent cardiac physiology. The condition of these individuals will most likely be unstable with filling and draining [35, 48], and they may require tidal PD prescriptions or an alternative modality. Indeed, the beneficial aspects of PD (slow solute clearance and ultrafiltration) also become one of its disadvantages for several patient populations, particularly those with severe fluid overload or severe lactic acidosis requiring precise fluid balance and controlled ultrafiltration that can only be attained with IHD or CRRT.

PD has limitations for certain populations. Those with pulmonary compromise may have a worsening of their symptoms due to increased abdominal dialysate volumes, thereby preventing full diaphragmatic excursion [60, 61]. Patients with ventriculoperitoneal shunts or prune belly syndrome have been successfully dialyzed with PD but do present increased potential complications. Finally, patients that have undergone abdominal surgery may not be amenable to utilizing this therapy, and, in patients with diaphragmatic defects, the use of PD is contraindicated. The process of PD itself can cause significant losses in immunoglobulins, increasing the risk of infections in these patients. Peritonitis can lead to further increased dialysate protein loss, nutritional compromise, loss of ultrafiltration capacity, and permanent damage to the peritoneal membrane [62].

Mechanical complications associated with PD include leaks, hernias and catheter obstruction. Dialysate leakage may occur around the catheter or into the pleural space, resulting in hydrothorax. Drainage problems seen with PD are typically caused from catheter malfunctions in the form of omentum and fibrin clot obstructions [48, 50]. The potential for complications from infections must not be overlooked, and the clinician must remain vigilant to the possibility of peritonitis, particularly fungal peritonitis.

Manual PD can be labor intensive for the bedside nurse, depending on frequency of cycles. Additionally, warming of dialysate is difficult without the use of an automatic cycler. In the critical-care setting, warming of PD solution is often difficult and overlooked, yet it is an important component for maintaining hemodynamic stability and improving effective solute clearance. Overall, the largest expense associated with PD, outside of nursing, is the cost of the dialysis fluid itself, regardless of whether it is commercially prepared or “custom-made”.

#### The case for IHD in pediatric AKI

IHD has the clear advantage of rapid ultrafiltration or solute removal when compared to PD or CRRT. In the hemodynamically stable patient, no RRT modality is better suited then IHD for the rapid clearance of an offending solute. Along with PD, IHD can be performed outside of an ICU setting. This method of therapy is particularly important in ingestions, drug toxicity, tumor lysis syndrome and hyperammonemia seen in the pediatric population [24, 41, 42]. In such cases, CRRT can be utilized as step-down therapy to prevent a rebound of certain toxins (i.e. ammonia, lithium) once the initial IHD run has been completed [41, 43]. The ability to adjust dialysate composition to treat various electrolyte abnormalities (i.e. hyperkalemia, hypernatremia) quickly is a major advantage when compared to PD or CRRT [63].

### Technical aspects

IHD requires technical expertise on the part of the physician, nurse and technician for optimal support for the wide range of pediatric patients, and, typically, it is available in larger secondary and tertiary care hospitals. Vascular access is, indeed, arguably the most important component contributing to the satisfactory provision of this therapy. In neonates the use of umbilical veins is an important consideration for vascular access. Utilization of neck veins, although limited by anatomy, may be preferable, especially in infants weighing less than 5 kg. Such access may allow for less recirculation and avoid the potential high venous return pressures often associated with groin lines in patients with high intra-abdominal pressures. While difficult in the smallest infants, it is possible. A wide variety of temporary vascular catheters are available for the pediatric population [64]. The placement of vascular access can be performed at the bedside by pediatric nephrologists or intensivists, for short-term catheters, or in an operating room by a surgeon or an interventional radiologist for permanent tunneled catheters. Placement of temporary or permanent vascular catheters for IHD can result in blood vessel stenosis or thrombosis, along with air emboli or hemorrhage. Depending on location or degree of difficulty involved in the placement of short-term or permanent vascular catheters, future access needs may be compromised. This issue becomes of great importance for patients requiring long-term access that progress from acute to chronic kidney injury. If possible, subclavian catheter placement should be avoided altogether, due to the very high incidence of stenosis at the puncture site, which may render the formation of a fistula impossible in the future. IHD can be utilized without anticoagulation and does not require the extra central line placement often needed for CRRT regional anticoagulation with citrate.

### Efficiency and therapeutic advantages/disadvantages

Because of the advantage of rapidity of fluid and/or solute removal afforded by IHD, dialysis disequilibrium is a potential complication seen in patients receiving this therapy. Careful dosing, dialysate solution selection, judicious use of mannitol, and monitoring are required to prevent the dangerous osmolar shifts that can cause complications of mental status changes and/or seizures produced from the effects of cerebral edema [65]. Caution must also be employed in the treatment of non-uremic patients (e.g. inborn errors or intoxications). These patients are at risk for developing severe hypophosphatemia, and their levels of serum phosphate (PO_4_) must be carefully monitored. Phosphate supplementation may be accomplished by addition to the dialysate bath (concentration from 0.5 mmol/l to 1.5 mmol/l, as needed) or by the administration of a separate phosphate infusion. These potential complications reinforce the belief that IHD should only be prescribed by nephrologists.

Additionally, as IHD will be able to achieve only a certain amount of ultrafiltration in the time available for treatment, fluid restriction usually will be required in the oliguric or anuric AKI patient. This can limit the amount of nutrition a patient will be able to receive over a 24-hour period. Hypotensive patients will have a limited ability to achieve appropriate fluid removal or even tolerate IHD. In such cases PD or CRRT may provide a more gradual ultrafiltration and be better tolerated. Filter membrane biocompatibility (BCM) vs bioincompatibility (BICM) appears to be an unresolved issue at this time. Certain authors have suggested that only biocompatible membranes be used in children with AKI [35], but a large Cochrane review concluded that there is no demonstrable clinical advantage to the use of BCM versus BICM in patients (> 18 years of age) with acute renal failure (ARF) who require IHD [66].

#### The case for CRRT in pediatric AKI

CRRT is beginning to emerge from its infancy in terms of when and how it should be used, which patient population will most benefit, and which complications are most commonly seen. Over the past 5 years several defining principles of CRRT in infants and children have been established. This work has been spear-headed by a pediatric-based consortium comprising 13 US centers. This group, known as the prospective pediatric CRRT (ppCRRT) consortium, has redefined the demographics, outcomes, and anticoagulation strategies, among other important variables, in the treatment approach to pediatric AKI [23, 65–71]. Perhaps, one of the most important variables has been the degree of fluid overload patients have when CRRT is initiated, and its relationship to outcome. There are now four published reports pointing out the importance that the degree of fluid overload plays in the management of pediatric AKI [71–74]. With these recent developments, it appears as though changes in our treatment of the sickest patients with CRRT has resulted in an improvement in stratification of mortality rates compared to historical outcomes [71, 75].

### Technical aspects

Perhaps one of the greatest drawbacks of CRRT is its complexity and expense compared with those of PD and, to a lesser extent, IHD [35]. The new machinery available for CRRT has resulted in improved safety, but it has also increased costs. The safe and proper provision of CRRT requires specialized nursing education and pharmacy support. Many institutions have compounded CRRT solutions; however, with recent approval by the Food and Drug Administration (FDA) of both commercially available dialysate and filter replacement solutions, these support requirements have substantially decreased [76–78].

There are also several unique requirements for CRRT implementation in pediatrics. As with IHD, adequate vascular access is required for CRRT. This technical aspect is the most important consideration contributing to the effectiveness of the therapy. Depending on the type of anticoagulation used, an additional central line may be required, as with regional citrate anticoagulation.

Historically, arteriovenous circuits were utilized; however, these have given way to commercially available venovenous therapies, which are pump driven and provide more predictable blood flow and, therefore, solute and ultrafiltration rates. Despite the availability of smaller circuits in some jurisdictions, the large extracorporeal volume required for CRRT and IHD (particularly in neonates) is a distinct disadvantage, necessitating blood priming in patients weighing less than 10 kg. In these settings, extracorporeal blood volume exceeds 10% of the patient’s blood volume. This exposes the patient to obvious risks associated with blood products and hemodynamic instability related to the flow of blood out of the body. Patients with multiple organ failure and hemodynamic instability may not tolerate the rapid circulation of blood through a CRRT circuit, regardless of the patient’s size [79]. Additionally, depending on the types of hemofilters utilized, hemodynamic instability may be observed with CRRT due to bradykinin reactions, activation of complement-coagulation cascade-monocytes, neutrophil degranulation and/or the release of reactive oxygen species [79, 80]. Some of the more significant technical disadvantages of CRRT include the requirement for continuous anticoagulation [69] and the clotting of circuits. While many easy anticoagulation protocols are available, anticoagulation comes with its own set of complications.

### Efficiency and therapeutic advantages/disadvantages

CRRT has several distinct advantages over IHD and PD in the treatment of patients with AKI. CRRT mimics the effect of renal function with its continual ultrafiltration and solute clearance [28, 30]. The predictability and efficiency of ultrafiltration (UF) and solute removal make CRRT ideally suited for the provision of RRT in hemodynamically unstable patients. A particular advantage afforded by CRRT (and IHD) is the ability for ultrafiltration to be separated from solute removal, which allows more flexibility for prescriptions to be tailored to the patient’s needs.

Minimal requirements for fluid restriction, due to predictable and continual fluid removal while CRRT is being performed, allow improved and adequate nutritional delivery. The need for supplemental protein (as high as 3–4 gm/kg per day) during CRRT must not be under-estimated when this therapy is being used, as the sieving coefficients of most amino acids are close to 1, and, therefore, clearance is quite high and can result in a nutritional deficit [81].

In the setting of AKI, CRRT can rapidly and predictably restore homeostasis. Indeed, CRRT provides superior uremia control compared to PD [49, 82] or even intermittent daily hemodialysis [83, 84]. Alteration of dialysate or filter replacement fluid is possible and can allow the clinician to control electrolyte levels within a desired target range; this is particularly important in the setting of increased intracranial pressure, when higher sodium levels may be required or when hyperosmolar states need to be corrected [85]. Of course, due to potential compounding errors, caution needs to be exercised when these alterations are being made, and they should be done under careful guidance of the clinician and pharmacist [77].

CRRT during AKI may have the additional benefit of restoring immuno-homeostasis via removal of both pro-inflammatory and anti-inflammatory molecules [86]. This remains an area of active ongoing research.

### CRRT vs PD and IHD outcomes

At the present time there have been no randomized clinical trails comparing PD vs IHD vs CRRT for treatment of children with AKI. To date only one randomized clinical study of adults, comparing CRRT with PD, has been published. This study was performed in a developing country in terms of resources. Phu et al. [87] made an open randomized comparison of PD vs CRRT in patients with infection-related AKI. This 70-patient study (*n* = 34 on CRRT, *n* = 36 on PD) found that for all their primary end points (i.e. resolution of acidosis, reduction of creatinine), CRRT was significantly superior to PD. Additionally, they noted as secondary outcomes a significant survival difference between modalities, with CRRT resulting in an 85% survival rate and PD associated with a 53% survival rate. They also noted an overall cost reduction for patient care with the use of CRRT, despite the higher technical costs of this therapy. This was due to savings in duration of therapy and overall reduction in total resource requirements per patient. Their overwhelming conclusion was that CRRT was superior to PD for the treatment of AKI associated with infection. Their study has been criticized for the prescription delivered with PD and other methodological issues leading to potential selection bias [87, 88], but, nonetheless, the study provides the first randomized comparison of these modalities. More recently, a pediatric-based retrospective analysis [49] of 118 infants and children treated either with PD (*n* = 82) or CRRT (*n* = 36) (followed by extended daily dialysis in those showing signs of recovery) demonstrated that, while there was no difference in mortality rates between modalities, CRRT provided better fluid control and was the modality of choice for hypercatabolic AKI associated with sepsis. Main CRRT complications were access and circuit clotting. The patients’ conditions and modality choices were quite different, with smaller patients with more stable conditions receiving PD, while hemodynamically unstable, somewhat larger, patients received CRRT. These data support, in part, the conclusions reached by Fleming et al. [82]. They retrospectively compared PD (*n* = 21) and CRRT (*n* = 21) in 42 children following repair of congenital heart disease lesions. The common indications for RRT implementation included fluid overload, electrolyte abnormalities, provision of total parenteral nutrition (TPN), and oliguria. No standardized initiation criteria were utilized, and this varied significantly among patients. Additionally, nine patients in the CRRT group received arteriovenous CRRT. Most of the patients (90%) required pressor support. While there was no difference noted in terms of mortality rate between modalities (62% for both), CRRT was superior to PD for ultrafiltration, solute clearance and nutritional provision. From these data, the authors concluded that CRRT was superior to PD in this clinical setting. The conclusions of their study must be guarded, as patient care was not standardized in terms of modality initiation criterion. Also, the patient group was rather homogeneous, making generalizability difficult, and no apparent survival benefit was noted with improved solute and ultrafiltration in the CRRT group. Bunchman et al. [75] reviewed survival outcome in 226 pediatric patients receiving various forms of RRT, including PD, IHD and CRRT, over a 7-year period from 1992–1998. Patients were treated with CRRT (*n* = 106), IHD (*n* = 61) or PD (*n* = 59). Factors influencing patient survival included: (1) low blood pressure (BP) at the initiation of RRT (33% survival, low BP; 61%, normal BP; 100%, high BP; *P* < 0.05); (2) pressor use anytime during RRT (35% survival on pressors; 89% survival not requiring pressors; *P* < 0.01); (3) diagnosis (improved outcome in those with primary renal failure compared to those with secondary renal failure; *P* < 0.05); (4) RRT modality (40% survival, CRRT; 49% survival, PD; 81% survival, IHD; P < 0.01 IHD vs PD or CRRT), and (5) pressor support was significantly higher in children on CRRT (74%) and PD (81%) vs IHD (33%) (*P* < 0.05 IHD vs CRRT or PD). The authors concluded that hemodynamic support (sicker patients) with pressors imparted a greater prediction of mortality, rather than RRT modality, and that survival of children, as of adults, is best predicted by the underlying diagnosis and hemodynamic stability. Interestingly, modality choice was determined, in part, by patient status. That is, patients with greater hemodynamic instability were preferentially treated with PD or CRRT, and many of these patients required pressor support.

## Discussion

The data for RRT modality choice in the treatment of pediatric AKI are clearly limited. As in many areas of pediatrics, the majority of reports are retrospective, small, non-randomized, single-center studies that reflect a homogeneous group of patients and have limited scope. Indeed, many of these studies inherently reflect the practice standards of the institution and investigator bias. There are several questions that clinicians must address when making RRT modality decisions for treating pediatric AKI: “When should we initiate dialysis or how aggressive should volume and solute be controlled?”; “How severely ill is the patient and what modality would best serve the patient’s needs/limitations?”; “What modalities are available?”; “What is the local expertise for delivering the modality?”

In order to address these questions we must develop standard principles to allow comparative analysis of pediatric patients with AKI. Foremost is the acceptance of common criteria for the diagnosis of AKI. With the recent validation of the pRIFLE criteria [6], we have a consensus definition that could, for the first time, allow us to compare data sets across institutions. This could revolutionize our approaches to RRT modality choice and, indeed, guide future randomized studies. While the indications for dialysis are reasonably well understood, data regarding when to intervene with RRT are limited. Through prospective multicenter analysis in pediatrics, the ppCRRT consortium has nicely demonstrated a variety of important variables in terms of treatment of pediatric AKI, including fluid overload as an independent risk factor for mortality [23, 67–71]. These data, along with data from adults [89], suggest that early intervention is essential for the improvement of outcomes in terms of CRRT. Indeed, earlier intervention with PD in post-operative children with congenital heart disease has also been proposed to have survival advantages [90]. These data support the use of early intervention prior to significant (> 15% over body weight) fluid overload [71–74]. In addition to these variables, the particular role of “dosing of dialysis” has become an increasingly important variable. “Dialysis dose” disparity between CRRT, IHD and PD makes it difficult for them to be compared adequately with each other in a meaningful fashion. Indeed, even intra-modality variability of dosing is the subject of investigation. Ronco et al. [89], in their landmark randomized study of adults on CVVH, demonstrated that an ultrafiltration prescription (dose) of 35 ml/kg per hour was superior to that of 20 ml/kg per hour in terms of overall patient survival. Furthermore, subgroup analysis revealed that patients with sepsis benefited when higher ultrafiltration rates (45 ml/kg per hour) were utilized. No such dosing studies have been performed in pediatric patients, and most centers have used a rate of 2,000 ml/1.73 m^2^ body surface area per hour (an extrapolation based on IHD), with a minimum rate of 35 ml/kg per minute.

Also evident in the literature review is the necessity of use of a severity of illness score when a patient’s clinical status is being evaluated. Clearly, false conclusions can be made if sicker patients are included in one modality vs another. This has been demonstrated by Swartz et al. [84, 91] in two analyses. In the first [91], 350 adult patients with AKI were treated with either IHD or CRRT. This retrospective analysis suggested that patients treated with CRRT had poorer outcomes; however, when multivariate analysis was used to control for severity of illness, CRRT and IHD appeared to have equivalent outcomes. In the second [84], more recent, study of 222 patients from the same center, it appeared that, when severity of illness was controlled for, CRRT was superior to IHD in terms of patient survival. Pediatric studies have also demonstrated the necessity for adaptation of severity of illness scores for the adequate comparison of RRT modality efficiencies [92, 93].

In centers with limited resources, the onus is on optimizing and implementing available care, as was nicely outlined by Phadke and Dinaker [50] and Ronco [94]. With further clarification of variables affecting outcome in pediatric AKI, the mortality rate should be affected in a positive fashion. The epidemiology and technical expertise available for treatment of pediatric AKI will continue to evolve. In order to serve the pediatric population better, the clinician’s responsibility is to utilize the literature and updated developments to guide our choices. Indeed, practice standards for AKI RRT management have been proposed in Europe, based on available data and consensus [79]. The challenge facing the pediatric nephrology and intensivist community in developed countries is to carry out multi-center, randomized, controlled trials comparing patients with similar stages of AKI stratified to the various RRT modalities. We have come close to being able to carry out such studies with the availability of tools such as the pRIFLE criteria, severity of illness scores, and identifiable important variables (i.e. fluid overload). In addition to these important developments, the ongoing identification and predictive validation of AKI biomarkers [95–99] in terms of onset, course and prognosis of pediatric AKI makes the coming years very exciting.

## Questions

(Answers appear after the reference list)

1. The use of temporary peritoneal dialysis would be the last choice in terms of provision of renal replacement therapy in a patient with acute kidney injury in which setting?
  - A 4-year-old patient with *E. coli*-associated hemolytic uremic syndrome, with mild gastrointestinal symptoms and anuric acute kidney injury
  - A 14-year-old girl with mild (< 5% over body weight) fluid overload, no pulmonary edema and a diagnosis of post-infectious glomerulonephritis with associated oliguria and acute kidney injury
  - A neonate which a status of post-operative repair of a ventricular septal defect (VSD) with hemodynamic instability, anuria and associated acute kidney injury presumed to be from acute tubular necrosis
  - A neonate with hyperammonemia secondary to an inborn error of metabolism presenting with acute kidney injury and dehydration
2. CRRT as a therapy offers which benefits over short-term PD?
  - It is widely available in developed and developing countries
  - It is technically easier to perform
  - It allows more accuracy in ultrafiltration and better solute clearance
  - Anticoagulation is not required
3. From the following dialytic indications, which would most fit with the choice of CRRT over PD in the setting of AKI? Choose all that apply.
  - Severe fluid overload (approximately 20% over base weight), pulmonary edema, hemodynamic instability, hyperphosphatemia and hyperkalemia
  - Neonate status post-operative. Left hypoplastic heart repair with compromised vasculature, hemodynamic instability, nominal fluid balance, normal electrolyte levels
  - A 16-year-old male trauma victim (automobile accident) with significant crush injury (myoglobinemia, hyperkalemia and hyperphosphatemia), status post-splenectomy
  - A bone marrow transplant (BMT) patient with sepsis and early fluid overload (10% over base weight) with hemodynamic instability, normal metabolic profile, anuria and the initial phases of pulmonary edema (not intubated yet).
4. The conservative management goals of pediatric AKI include which measures? Choose all that apply.
  - Control of fluid balance
  - No significant change in dietary intake
  - Blood pressure control
  - Improvement of survival
5. When considering dialysate solutions for either acute PD or CRRT in the setting of AKI, which of the following statements are true?
  - Pharmacy-prepared solutions are always the best options for provision of dialysate for both PD and CRRT
  - Commercially available, government regulatory body-approved dialysate solutions are available for both PD and CRRT
  - Lactate-based dialysate solutions are optimal for all patients when either PD or CRRT is used
  - Under special patient circumstances, custom-made solutions may be appropriate for patients receiving either PD or CRRT
